# The Impact of Helminth Infection on the Incidence of Metabolic Syndrome: A Systematic Review and Meta-Analysis

**DOI:** 10.3389/fendo.2021.728396

**Published:** 2021-08-12

**Authors:** Claire Rennie, Ritin Fernandez, Sheila Donnelly, Kristine CY McGrath

**Affiliations:** ^1^School of Life Sciences, Faculty of Science, University of Technology Sydney, Sydney, NSW, Australia; ^2^School of Nursing, University of Wollongong, Wollongong, NSW, Australia; ^3^Centre for Research in Nursing and Health, St George Hospital, Sydney, NSW, Australia; ^4^Centre for Evidence Based Initiatives in Health Care a JBI Centre of Excellence, Sydney, NSW, Australia

**Keywords:** metabolic syndrome, type 2 diabetes mellitus, helminth infection, parasitic worms, helminth therapy

## Abstract

**Background:**

There are a growing number of publications that report an absence of inflammatory based disease among populations that are endemic to parasitic worms (helminths) demonstrating the ability of these parasites to potentially regulate human immune responses. The aim of this systematic review and meta-analysis was to determine the impact of helminth infection on metabolic outcomes in human populations.

**Methods:**

Using PRISMA guidelines, six databases were searched for studies published up to August 2020. Random effects meta-analysis was performed to estimate pooled proportions with 95% confidence intervals using the Review Manager Software version 5.4.1.

**Results:**

Fourteen studies were included in the review. Fasting blood glucose was significantly lower in persons with infection (MD -0.22, 95% CI -0.40- -0.04, P=0.02), HbA1c levels were lower, although not significantly, and prevalence of the metabolic syndrome (P=0.001) and type 2 diabetes was lower (OR 1.03, 95% CI 0.34-3.09, P<0.0001). Infection was negatively associated with type 2 diabetes when comparing person with diabetes to the group without diabetes (OR 0.44, 95% CI 0.29-0.67, P=0.0001).

**Conclusions:**

While infection with helminths was generally associated with improved metabolic function, there were notable differences in efficacy between parasite species. Based on the data assessed, live infection with *S. mansoni* resulted in the most significant positive changes to metabolic outcomes.

**Systematic Review Registration:**

Website: PROSPERO Identified: CRD42021227619.

## Introduction

The metabolic syndrome is a cluster of risk factors with a diagnostic requirement of abdominal obesity and the presence of two or more of the following criteria: dysregulated glucose and lipid homeostasis; high blood pressure; abnormal cholesterol levels; and/or insulin resistance (IR) (1, 2). Having these traits greatly increases a person’s risk of developing type 2 diabetes (T2D) or cardiovascular disease, with the risk increasing proportionally to that of the number of traits present (3). Thus, it is perhaps not surprising that the prevalence of T2D is increasing in parallel to that of increasing obesity (4). Currently at epidemic proportions globally, predictive modelling estimates that the incidence will further increase by 51% in 2045 (5).

The explanation for the close association between obesity and T2D was first provided as early as 1993 by Hotamisligil et al., who demonstrated a causal link between obesity-induced pro-inflammatory cytokines and the development of IR (6), an observation which has since been corroborated by many research groups, both in animal models and human patients (7–11). More specifically, it has been shown that in obese individuals the pro-inflammatory cytokines TNF and IL-6 are significantly increased as compared to their leaner counterparts. This excess of pro-inflammatory cytokines impedes normal insulin signaling by promoting serine rather than tyrosine phosphorylation of the insulin receptor substrate-1, preventing translocation of the GLUT4 glucose transporter into the cell, leading to the development of an insulin resistant state (12). To compensate for the cell’s inability to take up glucose, the β-cells in the pancreas increase their insulin output to try to maintain glucose homeostasis. This eventually leads to cellular exhaustion, the deterioration of the β cells, and subsequent hyperglycemia (13). Dysregulated glucose homeostasis eventually leads to the development of T2D.

Current therapies for T2D primarily aim to manage hyperglycemia rather than target the underlying inflammation. The two main pharmacological treatments for T2D are metformin and sulphonylureas (14). However, due to the many side effects, such as gastrointestinal disturbance and intolerance, many other secondary treatments are commonly added or substituted in the treatment regime (14). Recognition that inflammation is involved in the pathogenesis of T2D provides a rationale for testing pharmacologically-directed anti-inflammatory treatments. Salicylates have direct anti-inflammatory effects *via* the inhibition of NF-κB activation (15). By inhibiting IKKβ, a key activator in the NF-κB pathway, obesity induced IR was reversed (15). Additionally, salicylate activates AMPK resulting in the phosphorylation of ACC, the rate limiting step of fatty acid synthesis (16). Salsalate, a prodrug form of salicylate that is better tolerated than salicylates, has been shown to improve fasting glucose, reduce circulating free fatty acids and increase adiponectin in humans (17). Unfortunately, in this same study, tinnitus and headaches were observed side effects at higher dosage, and although these were absent in the lower dosage groups, these lower dosages showed reduced efficacy. Further, the treatments were required to be taken 2-3 times daily and had no lasting effects (17). Therefore, there remains a clinically unmet need for treatments that can improve glucose homeostasis and inflammation, while not causing adverse side effects.

Emerging evidence suggests that parasitic worms (helminths) have the potential to treat underlying inflammation as well as improve glycaemia (18–20). The human immune system has evolved over millennia to provide protection from pathogenic micro-organisms such as viruses and bacteria. However, despite also being considered as pathogens, the immune response to infection with helminths does not convey protective immunity. Instead, the presence of the parasite is tolerated, with infections lasting several decades. It has been suggested that this unexpected outcome developed because the classic antimicrobial immune response was ineffective against these large worms and if activated would result in collateral damage to host tissue (21). Equally, helminth parasites cause extensive tissue disruption due to their feeding and migrating. Therefore, mammalian hosts have adapted to respond to helminth infection with a regulatory phenotype of immune response which operates to encapsulate the parasites while simultaneously repairing tissue damage (21). Indeed, there is an inverse relationship between the prevalence of inflammatory diseases and endemic infection with helminths, whereby diseases mediated by a dysregulated immune response are far more common in industrialized countries (22).

Rather than a classic protective pro-inflammatory Th1 type immune response, which is mounted against micropathogens (viruses, bacteria and protozoa), the typical immune response to helminth parasite infection is the development of a potent anti-inflammatory, type 2 immune response (23–26). This response is characterized by the secretion of cytokines, such as IL-4, IL-5 and IL-13, by T cells and polarization of macrophages towards an anti-inflammatory M2 phenotype with concomitant suppression of M1 inflammatory macrophages and the consequential inhibition of pro-inflammatory Th1 and Th17 responses (25, 27). Experimental studies in mice have supported the notion that helminth infection could be harnessed to regulate obesity driven inflammation to inhibit the development of metabolic disease. Mice infected with parasitic worms consistently showed reduced body weight and improved glucose metabolism compared to their uninfected counterparts (28–32). The mechanism behind these positive outcomes was shown to be an increased infiltration of M2 macrophages to the adipose tissue in response to the parasitic infection which suppressed the chronic pro-inflammatory response associated with obesity and thus led to an improvement in IR. While there is no direct evidence for a similar role for Group 2 Innate Lymphoid Cells (ILC2), these cells are quickly and robustly activated following helminth infection and are fundamental in regulating barrier tissue homeostasis and in the initiation and enhancement of the Th2 immune response (33, 34). Conversely, during obesity, a reduction in the number of ILC2s in adipose tissue has been associated with increased inflammation (35). Reversing this loss of ILC2s, as would occur during helminth infection, in obese mice restores glucose tolerance and insulin sensitivity (36, 37).

Based on this evidence there is growing support for the idea that controlled infection with live parasitic worms offers a novel strategy for the treatment of metabolic disorders caused by the chronic inflammation induced by obesity. However, despite the multiple publications of epidemiological and experimental studies there has only been one previous meta-analysis of the data (19). While this examined available data in publications up to 2016, as a result of the eligibility criteria only four publications were included in the review and minimal comparators were assessed. Since then, there have been an additional twelve publications. Furthermore, an analysis of the impact that the species of worm has on the outcome, and whether there is difference between an active or previous infection, has never been determined. Therefore, the present study aims to apply these considerations to a new meta-analysis of published evidence to more fully evaluate the effect of helminth infection on metabolic outcomes associated with glucose metabolism in humans. With a clinical trial testing the efficacy of intestinal parasite worm infection for the treatments of T2D underway (ACTRN12617000818336) (38), such an updated critical review of the evidence is timely.

## Methods

This systematic review and meta-analysis was conducted according to the Preferred Reporting Items for Systematic Reviews and Meta-Analysis guidelines (PRISMA) (39, 40) and registered with PROSPERO, registration number CRD42021227619.

### Search Strategy

Publications were sourced from Web of Science, SCOPUS, PubMed, Ovid MEDLINE, Cochrane CENTRAL, and Embase and included all publications up to August 2020. Terms searched were: (helminth OR helminths OR soil transmitted helminths OR strongyloides OR strongyloidiasis OR ascaris OR trichuris OR hookworm OR *Necator americanus* OR *Ancylostoma duodenale* OR schistosomiasis OR schistosoma OR schistosome OR nematode) AND (diabetes OR type 2 diabetes OR insulin resistance OR insulin sensitivity OR glucose metabolism OR glucose tolerance OR metabolic syndrome) NOT (type 1 diabetes). There was some variation in search terms between databases, for example, if there were a maximum number of conjunctions that could be used. Search results were uploaded to Endnote and duplicates excluded. One investigator then screened titles and abstracts for relevant research articles.

### Study Selection

This review included any study type, with samples of any size and on people of any age. Review articles, non-human models, studies that focused on type 1 diabetes, studies whose outcomes were unrelated to clinical glucose metabolism, and study protocols were excluded. Papers published in English between 2015 and 2020 were included.

### Quality Assessment

Two investigators independently assessed the quality of the accepted papers using the Joanna Brigg’s Institute appraisal tool (41). This instrument scores 8 methodological items for cross-sectional studies, 10 items for case control studies and 11 for cohort studies. The items are scored 0 (no), 1 (unclear/not applicable), or 2 (Yes). The maximum obtainable scores were 16 for cross-sectional studies, 20 for case control studies and 22 for cohort studies (**Supplementary Tables 1–3**). Disagreements were discussed between the investigators until a consensus was reached.

### Data Extraction

One reviewer extracted relevant information from acceptable papers including study design, sample size, population details, recruitment process, helminth exposure, method of diagnosis of infection and metabolic outcomes. If data were reported in separate metrics, outcome data extracted were converted to a common metric so treatment effects could be estimated.

### Data Analyses and Statistical Methods

The pooled data are presented as mean and 95% confidence interval using the extracted mean and the standard deviation values. All analyses were conducted using the Review Manager Software version 5.4.1 (42). Heterogeneity of the data was evaluated by calculating the I^2^ index (43). If the I^2^ value was <50%, the fixed effects model (FEM) was be applied. Sub-group analysis was conducted based on the type of helminth parasite.

## Results

### Study Selection

**Figure 1:**
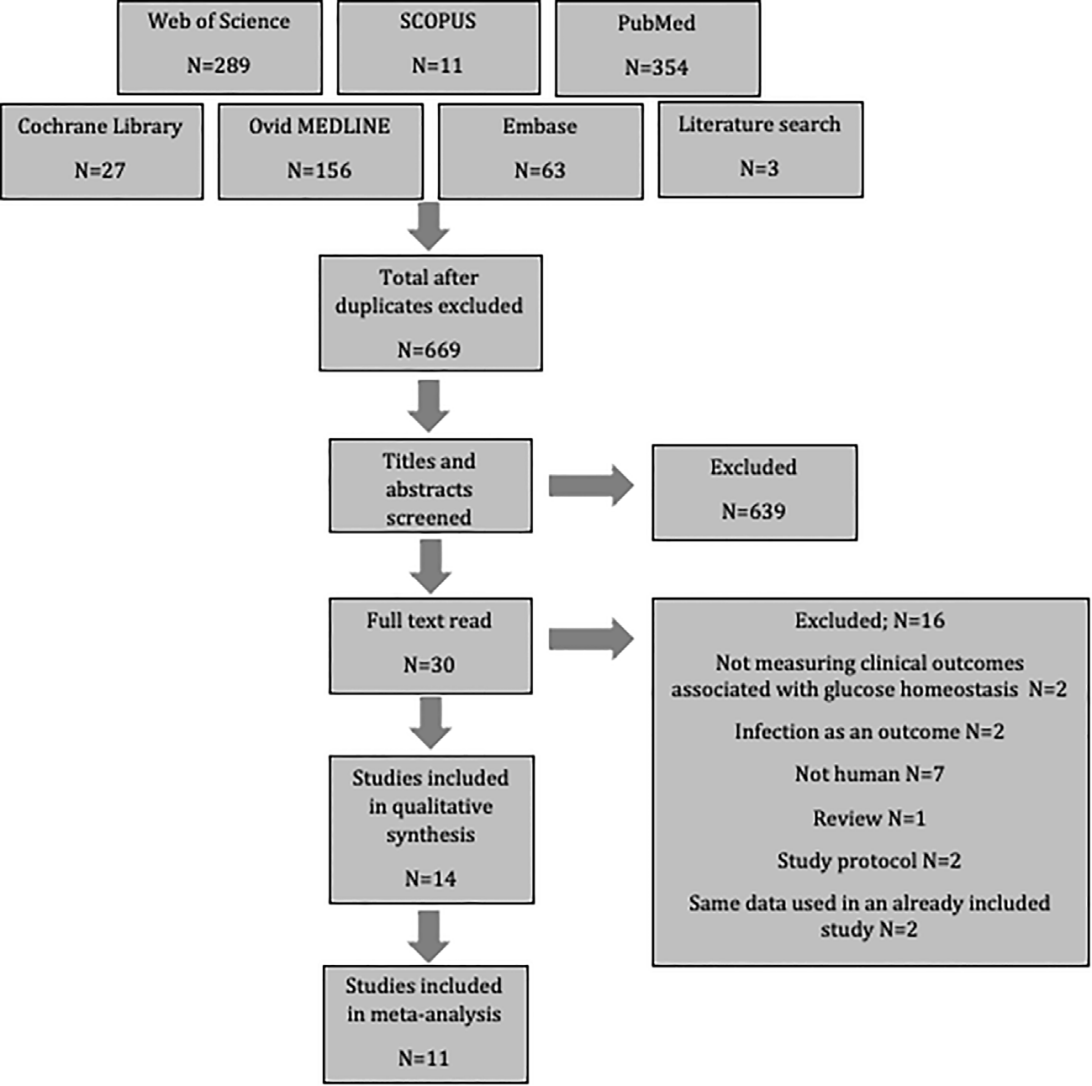
Flow diagram of study selection.

After searching six databases and exploring reference lists, 903 potential papers were identified. These were uploaded to Endnote, where duplicates were excluded. Titles and abstracts were then screened resulting in the removal of an additional 639 as they did not meet the inclusion criteria. A further 16 articles were excluded after full-text assessment revealed that they also did not meet the inclusion criteria; two did not measure outcomes associated with glucose homeostasis, two assessed infection as the only outcome, seven were not human populations, one was a review, two were study protocols, and two used the same population data as an already included study. This left fourteen studies for review. Three of these did not contain the standard deviation, and therefore could only be included in the systematic review, while eleven studies contained sufficient data to include in a meta-analysis (**Figure 1**).

### Quality Assessment

Thirteen of the studies were deemed to be of high quality as they scored ≥80% of the maximum obtainable score. One study (44) did not reach this cut off, so was not included in the meta-analysis, but was included in the systematic review.

### Study Characteristics

Of the fourteen studies included, ten were cross-sectional studies, two were case control, and two were cohort studies (**Supplementary Table 4**). These studies were conducted in Australia, China, India, Indonesia, England, Egypt, Ethiopia, Lao People’s Democratic Republic, South Africa, Thailand and Uganda. The sample sizes ranged from 158 to 9939, and ages ranged from 9 to 89.

Three of the studies investigated the effects on glucose homeostasis by previous parasite (Schistosoma spp.) infection, while eleven reportedly examined the impact of an active infection. Helminths assessed included *S. japonicum* [N=2 (44, 45)], *O. viverrini* [N=1 (46)], *S. stercoralis* [N=4 (47–50)], *S. mansoni* [N=1 (51)], Schistosoma spp. [N=2 (52, 53)] or any worm/multiple infection (*S. mansoni* or hookworm or *S. strongyloides* or *A. lumbricoides* or *T. trichura* or *E. vermicularis* or *S. haematobium* or *O. viverrini* or minute intestinal flukes or *Paragonimus* spp. or *S. stercoralis* or *cestodes/Taenia* spp.) [N=4 (26, 54–56)]. Infection was diagnosed by a variety of methods, namely stool examination/microscopy, serology or PCR (**Supplementary Table 4**).

The outcomes reported varied between studies, however all measured at least one marker relevant to glucose homeostasis. They included: fasting blood glucose (N=7), HbA1c (N=7), fasting plasma insulin (N=3), HOMA-IR (N=3), T2D diagnosis (N=7), and MetS diagnosis (N=3). For glycated hemoglobin (HbA1c), the studies included in this review used the DCCT (%) unit, where ≥6.5% was considered abnormal/diabetic. The MetS was characterized by central obesity and any two (45, 53) or three (52) of the following criteria defined by the International Diabetes Federation (2): (1) BP≥ 130/85mmHg or taking antihypertensive drugs; (2) TG≥150 mg/dl; (3) HDL-C <40 mg/dl in men, < 50 mg/dl in women; (4) FBG ≥100 mg/dl or taking hypoglycemic medications.

### Study Findings

Of the fourteen studies assessed, nine reported that infection with a parasitic worm had protective effects on diabetes-related parameters (26, 44–47, 50–53), however three studies (48, 49, 55) reported a positive association between specific infections and T2D diagnosis, and two studies (54, 56) reported no association. Individual glucose homeostasis related outcomes are summarized in **Supplementary Table 4**.

## Meta-Analysis of Selected Studies

### Association Between Parasite Infection and Fasting Blood Glucose

**Figure 2:**
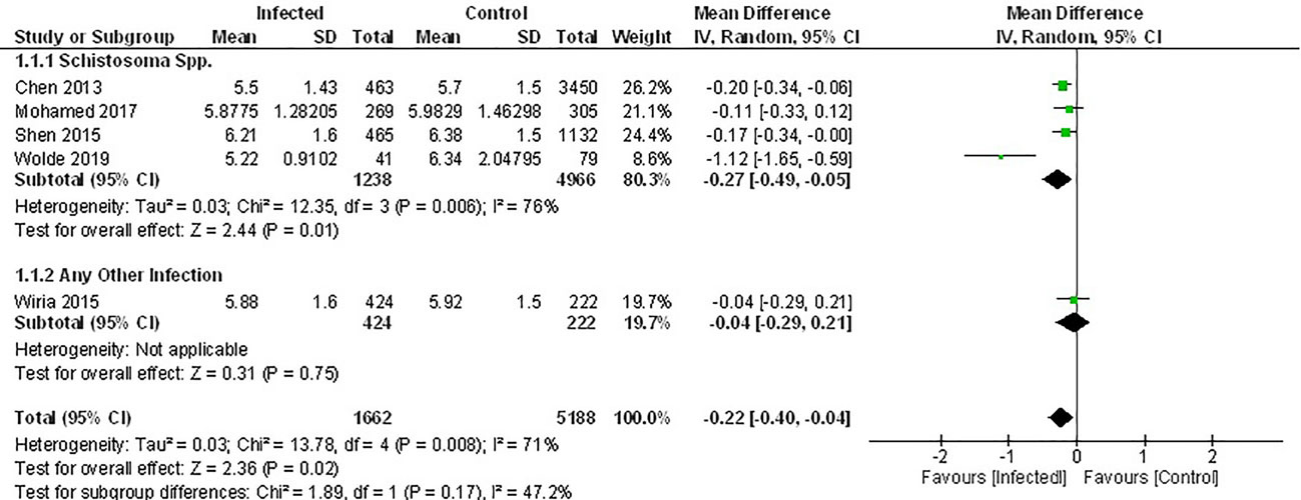
Effect of infection on fasting blood glucose. Forest plot summarising that of the 5 studies included herein, all report lower mean FBG levels in an infected population.

Of the seven studies that measured fasting blood glucose, only five provided sufficient data for inclusion in the meta-analysis. Pooled data from these studies demonstrated a significant decrease in FBG among those infected with parasites (**Figure 2**) (MD -0.22, 95% CI -0.40, -0.04; I^2 ^= 71%). However, subgroup analysis according to the genus of parasite revealed that infection with any soil-transmitted nematode had no effect on FBG (MD -0.27, 95% CI -0.49, -0.05; I^2 ^= 76%) compared to uninfected people. In contrast, there was a significant decrease in FBG among people infected with Schistosoma spp. (MD -0.27, 95% CI -0.49, -0.05; I^2 ^= 76%) compared to those not infected (**Figure 2**). Furthermore, the greatest beneficial impact was reported in people with active infections of *S. mansoni* (51) presented as the largest decrease in fasting blood glucose levels. In contrast, past infection with either *S. japonicum* (45) or *Schistosoma* spp (52, 53). had only a modest effect.

Results from two studies not included in the meta-analysis also demonstrated lower FBG among people chronically infected with *S. japonicum* (44), *S. mansoni* or hookworm (56) (**Supplementary Table 4**).

### Association Between Parasite Infection and HbA1c

**Figure 3:**
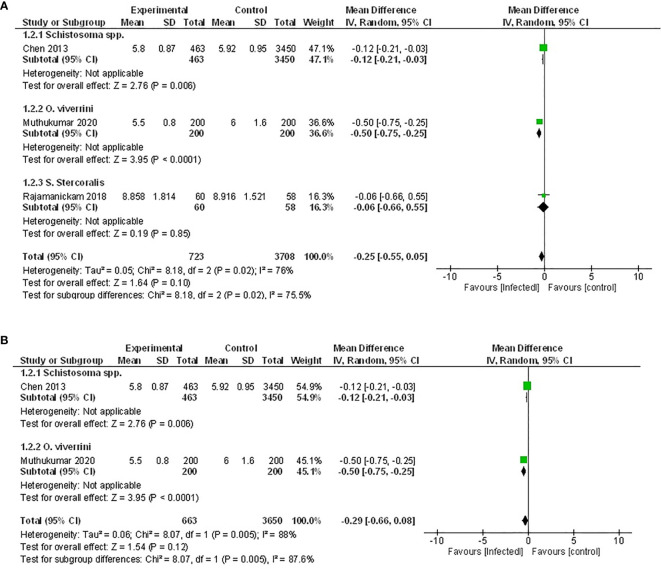
Effect of infection on HbA1c. **(A)** Forest plot of studies that measured HbA1c. Together, lower HbA1c is favored by infection. **(B)** Subgroup analysis excluding populations with diabetes only, infection favors lower HbA1c.

While six studies measured HbA1c, only three of those provided sufficient data for inclusion in a meta-analysis (46, 50, 52). Two of these studies examined the effect of infection with trematode parasites (46, 52), and one examined infection with an intestinal nematode (50). Either an active infection with *O. viverrini* (46) or a previous infection with *Schistosoma* spp (52). reported significantly lowered HbA1c. In contrast, the third study reported no effect when a population was actively infected with the intestinal nematode *S. stercoralis* (50) (**Figure 3**).

Pooled data from the three studies demonstrated no difference in HbA1c between infected patients and non-infected patients (**Figure 3A**, MD -0.25, 95% CI -0.55, 0.05; I^2^ = 76%), however, there was a trend towards lower HbA1c. As the presence of diabetes can impact the measure of HbA1c, the study in which the effect of helminth infection was measured in individuals with T2D (50) was excluded from a subsequent sensitivity analysis. The outcome of this was similar to the assessment of the pooled data set, with no difference in HbA1c between infected and non-infected people (**Figure 3B**, MD -0.29, 95% CI -0.66, 0.08; I^2^ = 88%).

Similarly, results from the remaining three studies not included in the meta-analysis also reported either no effect or a positive association between helminth infection and HbA1c (48, 54, 56).

### Association Between Parasite Infection and the Prevalence of the MetS

**Figure 4:**
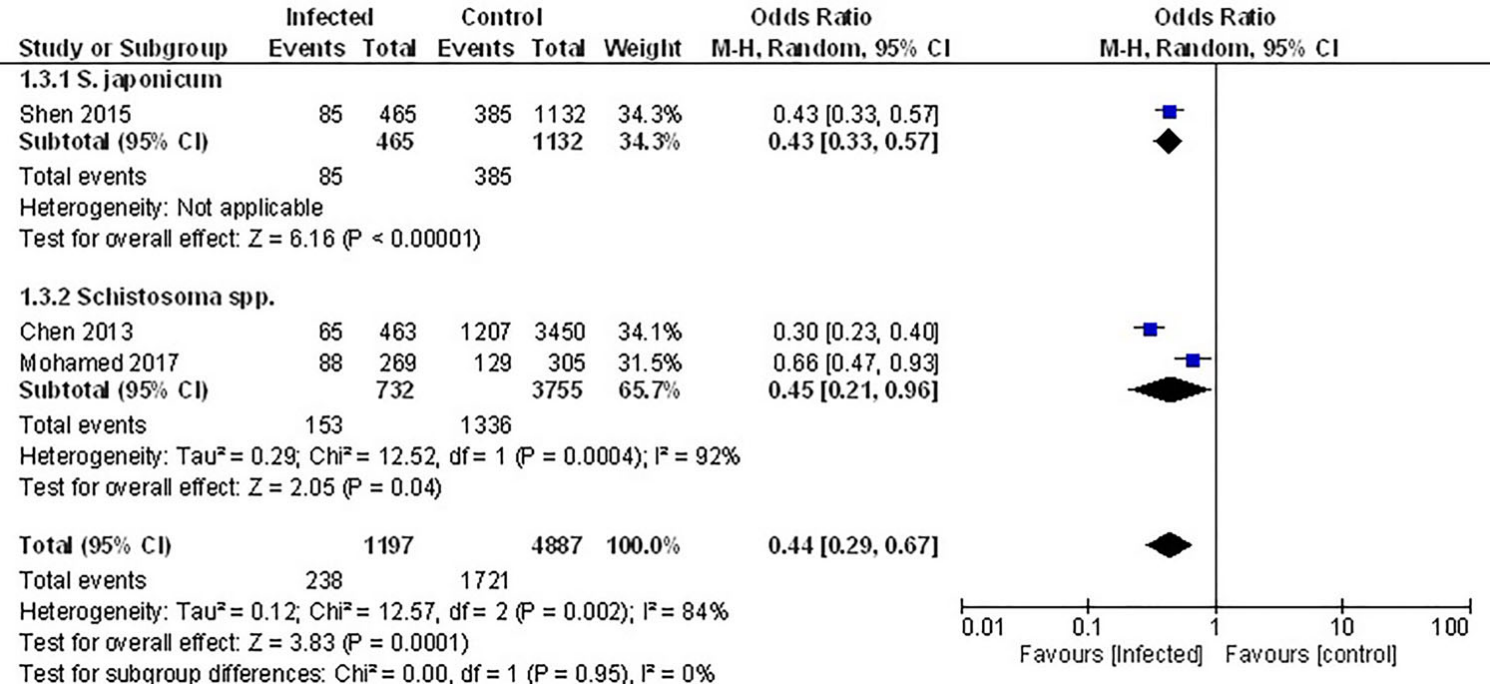
Effect of infection on the prevalence of the metabolic syndrome. Forest plot of studies which both individually and taken as a whole, demonstrate the protective effect of infection on prevalence of the MetS.

The only studies that examined the prevalence of the metabolic syndrome (MetS) were conducted in populations that had been previously infected with Schistosome parasites (45, 52, 53). Analyzing the pooled data from these, demonstrated that the prevalence of MetS was 56% lower among infected populations (**Figure 4**, OR 0.44, 95% CI 0.29, 0.67; I^2^ = 84%).

### Prevalence of Type 2 Diabetes in Infected and Non-Infected Populations

**Figure 5:**
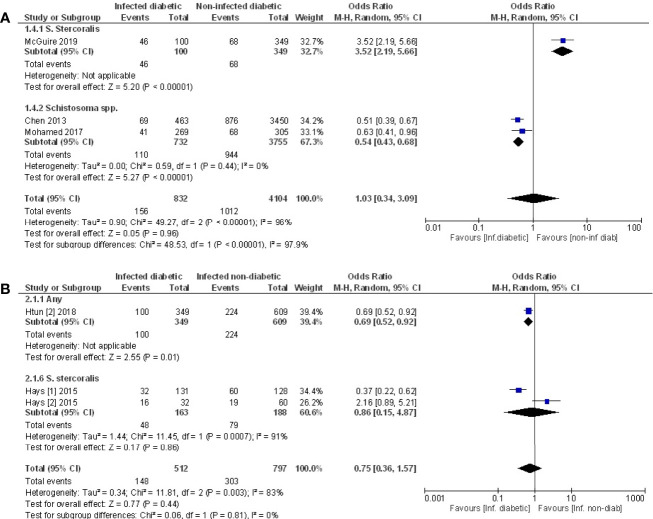
Prevalence of type 2 diabetes in infected or non-infected individuals. **(A)** Forest plot of studies illustrating that prevalence of T2D is not affected by infection when all studies are pooled. Subgroup analysis of only Schistosoma infection shows infection is associated with lower prevalence of T2D. **(B)** Forest plot of an infected population with diabetes *vs.* an infected population without diabetes shows people are less likely to develop diabetes if infected.

Pooled data from the three studies that compared the prevalence of T2D in infected *vs.* non-infected populations (49, 52, 53) demonstrated no difference in the prevalence of T2D (**Figure 5A**, OR 1.03, 95% CI 0.34, 3.09; I^2^ = 96%). However, sub-group analysis demonstrated that the prevalence of T2D was 46% lower among those infected with *Schistosoma* spp. (OR 0.54, 95% CI 0.43, 0.68; I^2^ = 0%).

### Prevalence of Infection in Populations With or Without Diabetes

Pooled data from three papers (47, 48, 55) that assessed prevalence of infection in populations with diabetes *vs.* without diabetes, a negative association between testing positive for infection and prevalence of T2D was evident (**Figure 5B**, OR 0.75, 95% CI 0.36, 1.57; I^2^ = 83%). The lack of significance likely reflects the variance in outcomes between the different studies. The two studies that examined people infected with *S. stercoralis* (47, 48) reported opposing effects while the third study (55) reported a negative association between any infection and T2D. This same study (55) reported a positive association of T2D with *Taenia* spp., however this cannot be included in the analysis because the populations with any infection are not discreet from the population where specific infections were assessed.

## Discussion

Increasing evidence indicates that human infection with parasitic worms has favorable effects on metabolic outcomes. Although recent experimental studies in mice have lent mounting support to these epidemiological observations, there is a lack of considered statistical assessment of the data. The aim of this study was to fill this gap in knowledge and critically evaluate the effect of helminth infection on metabolic outcomes within a broader human population.

While a previous meta-analysis concluded that helminth infection provided protection from metabolic dysfunction, this was based on only four studies up to 2016. This current review expands on the earlier analysis, as in addition to those four studies - 7 more recent publications were included in the meta-analysis. In addition to the larger sample sizes, our meta-analysis used a different approach to the previous study. Here, the data has been presented as mean values for all continuous outcomes, and the number of events for dichotomous outcomes. In contrast, the earlier meta-analysis treated every outcome as dichotomous and only presented the number of events. Furthermore, results in the previous analysis were initially presented as one single forest plot where multiple outcomes were combined, and the total numbers of events appear greater as they have added the total population for the studies each time when the study had multiple outcomes. However, a repeat of this analysis in which overlapping data sets were excluded (i.e. if one study analyzed multiple outcomes, only one outcome was included in the final analysis) did not reportedly create any substantial change in their results (19).

The outcomes from our meta-analysis were in agreement with the 2016 study and determined a generally positive effect of parasite infection on metabolic outcomes. However, it also revealed that not all parasite infections were equally efficacious. Direct comparison showed that infection with trematode flukes [*Schistosoma* spp. (45, 51–53) and *O. viverrini* (46)] had a greater effect on all measures compared to infection with intestinal nematode parasites [*S. stercoralis* (49, 50) and soil transmitted helminths (26)].

The reason for this striking difference is not obvious. As mentioned, the modulation of inflammation by all helminths has been characterized to be the same; a potent and biased Th2 immune response and a regulation of pro-inflammatory Th1 immunity, irrespective of the species of worm. However, from a host perspective, there are subtle differences in these immune responses. For the intestinal nematodes, the induction of Th2 cytokines leads to an increased turnover of intestinal epithelial cells and ultimately the expulsion of the worm (57). In contrast, for the tissue dwelling trematodes, the same type of immune response acts to repair tissue damage caused by the migrating worms and ultimately directs a tolerance towards the parasite, allowing the establishment of a long-term chronic infection (58, 59). However, in addition to this consideration, it is tempting to speculate that the positive effect of *Schistosoma* spp. and *O. viverrini* on the MetS may be due to the fact that these parasites have adapted to reside in the liver, thus directly targeting the organ that links obesity, IR and T2D (60).

An additional discovery from the current meta-analysis was a difference in efficacy between active and previous infections, with analysis suggesting that an active infection is the most beneficial. This is of interest as it has been hypothesized that the continued presence of helminth parasites may be required to elicit the regulatory effect on the human immune response (61–63).

This observation highlights the importance of correctly diagnosing the status of the parasite infection in order to make an accurate correlation with a therapeutic effect on a disease, as illustrated in the six studies that examined the effect of infection with *S. stercoralis.* Only three of these confirmed the presence of an active worm infection by detecting the presence of eggs in stool samples. In each case the parasite infection had no effect on metabolic outcomes (50, 54, 56). In contrast, two studies that reported a positive association with T2D and *S. stercoralis* infection used only seropositivity for anti-parasite IgG as a measure of parasite infection (47, 49). While this readout supports an exposure to the parasite, it does not necessarily indicate the presence of the parasite, and instead could suggest a past infection, which may be less likely to provide the necessary immune regulation to control obesity driven inflammation and thus MetS.

Equally, seropositivity, in the absence of eggs or larvae, may indicate a very recent infection or low dose, as proposed by Hays et al. (47, 48). In these studies, the population under examination were Aboriginal Australians from the Kimberly region, a location that is endemic for *S. stercoralis* (64, 65). One of these studies compared the prevalence of T2D in infected versus non-infected populations and found a negative association of T2D with seropositivity for the parasite, supporting the general hypothesis that infection with helminth parasites are beneficial (47). In contrast, a second study in the same community that compared the effect of parasite infection on T2D reported a positive association, with a higher number of people with T2D presenting with seropositivity for *S. stercoralis* compared to people without diabetes (48). A difference in the chronicity and the burden of parasite infections, and thus the extent of immune regulation may explain this apparent anomaly. For most helminth infections the switch of their host’s immune response towards the characteristic Th2/Treg phenotype can take time as it is often dependent on maturation of the parasite, the production of eggs and/or larvae, or simply the time it takes for the parasite to migrate to the tissue in which it will ultimately reside (66–68). In addition, it is acknowledged that the greater the worm burden, the stronger the immune regulation (69). Therefore, in the Australian population studied by Hays et al. (47, 48), it is possible that the population with reduced T2D are harboring a chronic and/or high dose parasite infection, which would have a more potent regulatory effect on the immune system. In contrast, the group of people in which there was no apparent beneficial effect, may have been recently infected and thus the regulation of immune responses by the parasite had not yet begun.

### Limitations

Unfortunately, due to the range in design and reporting of each of the studies, there are some limitations to the analyses presented here. The extent of the meta-analysis was restricted by the lack of consistency in the outcomes that were measured across different studies, thus reducing the number of data sets available for statistical comparison. Additionally, three studies were not added to the meta-analysis; two did not include standard deviation and the authors did not respond to requests for data, and one was the only study that measured the particular outcome, and so had no comparative data set for analysis.

Moreover, the conclusions made from this analysis regarding the impact of different parasites was based on the assumption that the data presented for single infections was arising from patients that had been screened for other pathogens. However, not all studies were explicit as to whether patients had been tested for concomitant infections. This is an important consideration as many of the populations represented in these studies are residing in regions that are endemic for multiple parasites, and indeed viral and bacterial pathogens, all of which will likely have some impact on the development of inflammatory mediated diseases, such as the MetS. Of the 14 studies examined for this review, four focused on populations where multiparasitism was evident, with some people carrying up to six different nematodes and trematodes (26, 54–56). In contrast to the other studies, where only a single parasite infection was evident, there was largely no change in any metabolic measures. Without detailed knowledge of each individual’s infection history, this is a very difficult dataset to interpret. Even if a person was apparently infected with only one parasite at the time of the study, the impact that previous infection with a different or multiple parasites has on their immune system, physiology and metabolism was not measured and therefore cannot be assessed. Thus, it is challenging to genuinely determine whether a specific parasite infection was mediating any effect on the development of MetS and/or T2D in this scenario. As mentioned above, the lack of consideration for worm burden and chronicity of infection hampered an exploration of the impact either of these factors have on the improvement of metabolic outcomes. Therefore, it is still unknown how quickly the helminth-induced effects take place and whether infection during early life is required to develop the immune-regulatory networks which mediate the therapeutic effect. Furthermore, the populations within these studies were all endemically infected with helminths. Such lifelong exposure ensures that a higher intensity of infection can be tolerated compared to naïve individuals not previously infected (70). Thus, for the purposes of translating the beneficial effect of parasite infection to treat MetS, a well-tolerated infection in non-endemic populations would require a lower worm burden, but this may reduce the potency and strength of the immune regulatory response (71, 72). Associated with that consideration, the conclusion regarding the beneficial effect of helminth infection would have been strengthened by the inclusion of some measure of inflammation, as this would have pointed to a mechanism that would inform future development of helminth-derived therapies. However, other than recording an increase in eosinophilia or seropositivity as a marker for parasite infection, there are no additional data regarding the effect of helminth infection on the underlying inflammation at the root of the MetS.

### Clinical Implications and Future Research

Currently the benefit of live helminth infection to individuals with central obesity and at least one MetS risk factor is being assessed in a double-blind, placebo-controlled clinical trial. Patients will be infected with 40 larvae of the human hookworm *Necator americanus* and changes in insulin sensitivity, body mass index and waist circumference will be measured over a 2-year period (ACTRN12617000818336) (38). Despite the limitations of our meta-analysis, the overall results clearly showed that infection with intestinal worms, such as hookworm, was less efficacious than the tissue dwelling parasites *S. mansoni* and *O. viverrini*. In all cases where an outcome could be compared, the impact of intestinal parasites was underwhelming, with no effect on HbAIc (50, 54, 56) or HOMA-IR (26, 56), only a modest effect on fasting blood glucose (26), and except for one study (47), either no effect or an increase in T2D (48, 49). In contrast, for every outcome, infection with *S. mansoni*, resulted in a significant positive effect (51).

The choice of hookworm for a clinical trial is based primarily on the lack of pathology that results from infection with this parasite. Other than a mild itch as the larval worms enter the body through the skin, for doses of up to 40 larvae, no discomfort has been reported in previous human safety trials (73–75). This parasite resides in the intestine of its human host where it attaches to the intestinal epithelium and feeds on blood. For a low dose/controlled infection, this has little pathological consequence, unlike with high doses where intestinal hemorrhage and iron deficiency anemia are common (76). Although *S. mansoni* also infects its human hosts through the skin, it resides in the mesenteric blood vessels, where the adult male and female worms mate to produce eggs that are then excreted from the body in order for the life cycle of the parasite to continue. While infection is rarely fatal, it can result in significant morbidities and loss of quality-of-life (77). Eggs that are not correctly secreted from the body can become trapped in the liver leading to the formation of granulomas and fibrosis which can result in portal hypertension and congestive splenomegaly (78). Liver enlargement and periportal fibrosis are commonly associated with advanced chronic infection. Children that are repeatedly infected can develop anemia and malnutrition which lead to significant developmental defects. Such outcomes clearly preclude the consideration of this parasite for live helminth therapy.

If live infection is deemed to be too high risk for a therapeutic intervention, there may be potential in using schistosome-derived products instead. Multiple experimental studies in murine models of obesity have tested the possibility that compounds derived from parasites can mimic the regulation of immune responses seen during live infection and thus exploited as therapeutics for inflammatory based diseases such as T2D. The most commonly investigated derivatives have been soluble egg antigen (SEA) from either *S. mansoni* or *S. japonicum*, and subsequently glycan and glycoproteins found within SEA, such as the Lewis^X^ containing Lacto-N-fucopentaose III (LNFPIII). The SEA, whether from *S. mansoni* or *S. japonicum*, consistently shows efficacy in models of IR and T2D, improving insulin sensitivity and glucose tolerance, increasing adipose M2 macrophages, increasing expression of IL-4 and -5, and increasing T regulatory cells (29, 79–82). Similarly, LNFPIII has also been shown to reduce white adipose tissue inflammation and improve adipose tissue insulin sensitivity (83). In addition to these secreted products, a myriad of immune-regulating molecules secreted by *S. mansoni* and many other helminths have been identified. Based on their varying mechanisms of action many of these also offer great potential to suppress obesity-driven inflammation (84, 85).

Combining these observations with the current meta-analysis, strongly support the proposal that helminth parasites have the capacity to regulate obesity driven inflammation to mediate a positive effect on metabolic outcomes. However, consideration for the variations between different parasites and a deeper understanding of the mechanisms involved is required before helminth-based therapies can progress to the clinic. This advancement would be greatly supported if future studies in the field included an accurate diagnosis of the parasite infection, information on the immunological and inflammatory status of patients, and consistent measures of metabolic outcomes. As this type of information is expanded and underscored with enhanced knowledge of the biochemistry and function of parasite-derived molecules, there is every possibility that helminth-derived therapy will be a clinical reality for patients with MetS.

## Data Availability Statement

The original contributions presented in the study are included in the article/**Supplementary Material**. Further inquiries can be directed to the corresponding authors.

## Author Contributions

CR conducted the search, identified the studies, performed the statistical analyses and wrote the first draft of this manuscript. CR and KM reviewed the quality of the studies. SD and KM conceptualized the study and supervised CR, RF, SD, and KM contributed to interpretation and revision of the manuscript. All authors contributed to the article and approved the submitted version.

## Funding

This research was supported by an Australian Government Research Training Program Scholarship.

## Conflict of Interest

The authors declare that the research was conducted in the absence of any commercial or financial relationships that could be construed as a potential conflict of interest.

## Publisher’s Note

All claims expressed in this article are solely those of the authors and do not necessarily represent those of their affiliated organizations, or those of the publisher, the editors and the reviewers. Any product that may be evaluated in this article, or claim that may be made by its manufacturer, is not guaranteed or endorsed by the publisher.

## References

[1] Internal Clinical Guidelines Team. Type 2 Diabetes in Adults: Management. London, UK: National Institute for Health and Care Excellence (2015). 26741015

[2] International Diabetes Federation. About Diabetes (2015). Available at: http://www.idf.org/about-diabetes.

[3] Sattar N McConnachie A Shaper AG Blauw GJ Buckley BM de Craen AJ. Can Metabolic Syndrome Usefully Predict Cardiovascular Disease and Diabetes? Outcome Data From Two Prospective Studies. Lancet (2008) 371(9628):1927–35. 10.1016/S0140-6736(08)60602-9 18501419

[4] Verma S Hussain ME. Obesity and Diabetes: An Update. Diabetes Metab Syndr: Clin Res Rev (2017) 11(1):73–9. 10.1016/j.dsx.2016.06.017 27353549

[5] Saeedi P Petersohn I Salpea P Malanda B Karuranga S Unwin N. Global and Regional Diabetes Prevalence Estimates for 2019 and Projections for 2030 and 2045: Results From the International Diabetes Federation Diabetes Atlas, 9th Edition. Diabetes Res Clin Pract (2019) 157:107843–53. 10.1016/j.diabres.2019.107843 31518657

[6] Hotamisligil GS Shargill NS Spiegelman BM. Adipose Expression of Tumor Necrosis Factor-Alpha: Direct Role in Obesity-Linked Insulin Resistance. Science (1993) 259(5091):87–91. 10.1126/science.7678183 7678183

[7] Kahn SE Hull RL Utzschneider KM. Mechanisms Linking Obesity to Insulin Resistance and Type 2 Diabetes. Nature (2006) 444(7121):840–6. 10.1038/nature05482 17167471

[8] Kern PA Ranganathan S Li C Wood L Ranganathan G. Adipose Tissue Tumor Necrosis Factor and Interleukin-6 Expression in Human Obesity and Insulin Resistance. Am J Physiol - Endocrinol Metab (2001) 280(5):E745–51. 10.1152/ajpendo.2001.280.5.E745 11287357

[9] Mohamed-Ali V Goodrick S Rawesh A Katz DR Miles JM Yudkin JS. Subcutaneous Adipose Tissue Releases Interleukin-6, But Not Tumor Necrosis Factor-α, in Vivo. J Clin Endocrinol Metab (1997) 82(12):4196–200. 10.1210/jc.82.12.4196 9398739

[10] Panagiotakos DB Pitsavos C Yannakoulia M Chrysohoou C Stefanadis C. The Implication of Obesity and Central Fat on Markers of Chronic Inflammation: The ATTICA Study. Atherosclerosis (2005) 183(2):308–15. 10.1016/j.atherosclerosis.2005.03.010 16285994

[11] Xu H Barnes GT Yang Q Tan G Yang D Chou CJ. Chronic Inflammation in Fat Plays a Crucial Role in the Development of Obesity-Related Insulin Resistance. J Clin Invest (2003) 112(12):1821–30. 10.1172/JCI200319451 PMC296998 14679177

[12] Draznin B. Molecular Mechanisms of Insulin Resistance: Serine Phosphorylation of Insulin Receptor Substrate-1 and Increased Expression of P85α. Two Sides Coin (2006) 55(8):2392–7. 10.2337/db06-0391 16873706

[13] Prentki M Nolan CJ. Islet β Cell Failure in Type 2 Diabetes. J Clin Invest (2006) 116(7):1802–12. 10.1172/JCI29103 PMC1483155 16823478

[14] The Royal Australian College of General Practitioners and Diabetes Australia. General Practice Management of Type 2 Diabetes. (2014-2015).

[15] Yuan M Konstantopoulos N Lee J Hansen L Li ZW Karin M. Reversal of Obesity- and Diet-Induced Insulin Resistance With Salicylates or Targeted Disruption of Ikkβ. Science (2001) 293(5535):1673–7. 10.1126/science.1061620 11533494

[16] Ford RJ Fullerton MD Pinkosky SL Day EA Scott JW Oakhill JS. Metformin and Salicylate Synergistically Activate Liver AMPK, Inhibit Lipogenesis and Improve Insulin Sensitivity. Biochem J (2015) 468(1):125–32. 10.1042/BJ20150125 PMC5233440 25742316

[17] Goldfine AB Silver R Aldhahi W Cai D Tatro E Lee J. Use of Salsalate to Target Inflammation in the Treatment of Insulin Resistance and Type 2 Diabetes. Clin Trans Sci (2008) 1(1):36–43. 10.1111/j.1752-8062.2008.00026.x PMC2662587 19337387

[18] Wiria AE Sartono E Supali T Yazdanbakhsh M. Helminth Infections, Type-2 Immune Response, and Metabolic Syndrome. PLoS Pathog (2014) 10(7):e1004140. 10.1371/journal.ppat.1004140 24992724 PMC4081794

[19] Tracey EF McDermott RA McDonald MI. Do Worms Protect Against the Metabolic Syndrome? A Systematic Review and Meta-Analysis. Diabetes Res Clin Pract (2016) 120:209–20. 10.1016/j.diabres.2016.08.014 27596058

[20] Moyat M Coakley G Harris NL. The Interplay of Type 2 Immunity, Helminth Infection and the Microbiota in Regulating Metabolism. Clin Trans Immunol (2019) 8(11):e01089–9. 10.1002/cti2.1089 PMC6837856 31719981

[21] Gause WC Wynn TA Allen JE. Type 2 Immunity and Wound Healing: Evolutionary Refinement of Adaptive Immunity by Helminths. Nat Rev Immunol (2013) 13(8):607–14. 10.1038/nri3476 PMC3789590 23827958

[22] Zaccone P Fehervari Z Phillips JM Dunne DW Cooke A. Parasitic Worms and Inflammatory Diseases. Parasite Immunol (2006) 28(10):515–23. 10.1111/j.1365-3024.2006.00879.x PMC1618732 16965287

[23] Infante-Duarte C Kamradt T. Th1/Th2 Balance in Infection. Springer Semin Immunopathol (1999) 21(3):317–38. 10.1007/BF00812260 10666776

[24] Pearce EJ Caspar P Grzych JM Lewis FA Sher A. Downregulation of Th1 Cytokine Production Accompanies Induction of Th2 Responses by a Parasitic Helminth, Schistosoma Mansoni. J Exp Med (1991) 173(1):159–66. 10.1084/jem.173.1.159 PMC2118762 1824635

[25] Pearce EJ Kane C Sun J Taylor J McKee AS Cervi L. Th2 Response Polarization During Infection With the Helminth Parasite Schistosoma Mansoni. Immunological Rev (2004) 201(1):117–26. 10.1111/j.0105-2896.2004.00187.x 15361236

[26] Wiria AE Hamid F Wammes LJ Prasetyani MA Dekkers OM May L. Infection With Soil-Transmitted Helminths Is Associated With Increased Insulin Sensitivity. PLoS One (2015) 10(6):e0127746. 10.1371/journal.pone.0127746 26061042 PMC4464734

[27] Allen JE Maizels RM. Diversity and Dialogue in Immunity to Helminths. Nat Rev Immunol (2011) 11(6):375–88. 10.1038/nri2992 21610741

[28] Berbudi A Surendar J Ajendra J Gondorf F Schmidt D Neumann AL. Filarial Infection or Antigen Administration Improves Glucose Tolerance in Diet-Induced Obese Mice. J Innate Immun (2016) 8(6):601–16. 10.1159/000448401 PMC6743339 27544668

[29] Hussaarts L García-Tardón N van Beek L Heemskerk MM Haeberlein S van der Zon GC. Chronic Helminth Infection and Helminth-Derived Egg Antigens Promote Adipose Tissue M2 Macrophages and Improve Insulin Sensitivity in Obese Mice. FASEB J (2015) 29(7):3027–39. 10.1096/fj.14-266239 25852044

[30] Su CW Chen CY Li Y Long SR Massey W Kumar DV. Helminth Infection Protects Against High Fat Diet-Induced Obesity via Induction of Alternatively Activated Macrophages. Sci Rep (2018) 8(1):1–14. 10.1038/s41598-018-22920-7 29545532 PMC5854586

[31] Wu D Molofsky AB Liang HE Ricardo-Gonzalez RR Jouihan HA Bando JK. Eosinophils Sustain Adipose Alternatively Activated Macrophages Associated With Glucose Homeostasis. Science (New York NY) (2011) 332(6026):243–7. 10.1126/science.1201475 PMC3144160 21436399

[32] Yang Z Grinchuk V Smith A Qin B Bohl JA Sun R. Parasitic Nematode-Induced Modulation of Body Weight and Associated Metabolic Dysfunction in Mouse Models of Obesity. Infect Immun (2013) 81(6):1905–14. 10.1128/IAI.00053-13 PMC3676010 23509143

[33] Löser S Smith KA Maizels RM. Innate Lymphoid Cells in Helminth Infections—Obligatory or Accessory? Front Immunol (2019) 10(620). 10.3389/fimmu.2019.00620 PMC6467944 31024526

[34] Bouchery T Le Gros G Harris N. ILC2s—Trailblazers in the Host Response Against Intestinal Helminths. Front Immunol (2019) 10(623). 10.3389/fimmu.2019.00623 PMC6458269 31019505

[35] Zeyda M Wernly B Demyanets S Kaun C Hämmerle M Hantusch B. Severe Obesity Increases Adipose Tissue Expression of Interleukin-33 and its Receptor ST2, Both Predominantly Detectable in Endothelial Cells of Human Adipose Tissue. Int J Obes (Lond) (2013) 37(5):658–65. 10.1038/ijo.2012.118 22828942

[36] Brestoff JR Kim BS Saenz SA Stine RR Monticelli LA Sonnenberg GF. Group 2 Innate Lymphoid Cells Promote Beiging of White Adipose Tissue and Limit Obesity. Nature (2015) 519(7542):242–6. 10.1038/nature14115 PMC4447235 25533952

[37] Hams E Locksley RM McKenzie AN Fallon PG. Cutting Edge: IL-25 Elicits Innate Lymphoid Type 2 and Type II NKT Cells That Regulate Obesity in Mice. J Immunol (2013) 191(11):5349–53. 10.4049/jimmunol.1301176 PMC3847854 24166975

[38] Pierce D Merone L Lewis C Rahman T Croese J Loukas A. Safety and Tolerability of Experimental Hookworm Infection in Humans With Metabolic Disease: Study Protocol for a Phase 1b Randomised Controlled Clinical Trial. BMC Endocr Disord (2019) 19(1):136. 10.1186/s12902-019-0461-5 31829172 PMC6907345

[39] Liberati A Altman DG Tetzlaff J Mulrow C Gøtzsche PC Ioannidis JP. The PRISMA Statement for Reporting Systematic Reviews and Meta-Analyses of Studies That Evaluate Healthcare Interventions: Explanation and Elaboration. BMJ (2009) 339:b2700. 10.1136/bmj.b2700 19622552 PMC2714672

[40] Moher D Liberati A Tetzlaff J Altman DG. Preferred Reporting Items for Systematic Reviews and Meta-Analyses: The PRISMA Statement. PLoS Med (2009) 6(7):e1000097. 10.1371/journal.pmed.1000097 19621072 PMC2707599

[41] Moola S Munn Z Tufanaru C Aromataris E Sears K Sfetcu R. “Chapter 7: Systematic Reviews of Etiology and Risk”. In: Joanna Briggs Institute Reviewer’s Manual (2020). p. 2019–05.

[42] Review Manager (RevMan) (Computer Program), Vol. Version 5.4.1 (2020).

[43] Higgins JP Thompson SG. Quantifying Heterogeneity in a Meta-Analysis. Stat Med (2002) 21(11):1539–58. 10.1002/sim.1186 12111919

[44] Duan Q. Population Based and Animal Study on the Effects of Schistosoma Japonicum Infection in the Regulation of Host Glucose Homeostasis. Acta Trop (2018) 180:33–41. 10.1016/j.actatropica.2018.01.002 29309743

[45] Shen SW. The Potential Long-Term Effect of Previous Schistosome Infection Reduces the Risk of Metabolic Syndrome Among Chinese Men. Parasite Immunol (2015) 37(7):333–9. 10.1111/pim.12187 25809087

[46] Muthukumar R. Effects of Opisthorchis Viverrini Infection on Glucose and Lipid Profiles in Human Hosts: A Cross-Sectional and Prospective Follow-Up Study From Thailand. Parasitol Int (2020) 75:102000. 10.1016/j.parint.2019.102000 31669292

[47] Hays R. Does Strongyloides Stercoralis Infection Protect Against Type 2 Diabetes in Humans? Evidence From Australian Aboriginal Adults. Diabetes Res Clin Pract (2015) 107(3):355–61. 10.1016/j.diabres.2015.01.012 25656764

[48] Hays R Esterman A McDermott R. Type 2 Diabetes Mellitus Is Associated With Strongyloides Stercoralis Treatment Failure in Australian Aboriginals. PLoS Negl Trop Dis (2015) 9(8):e0003976. 10.1371/journal.pntd.0003976 26295162 PMC4546619

[49] McGuire E Welch C Melzer M. Is Strongyloides Seropositivity Associated With Diabetes Mellitus? A Retrospective Case-Control Study in an East London NHS Trust. Trans R Soc Trop Med Hyg (2019) 113(4):189–94. 10.1093/trstmh/try132 30597107

[50] Rajamanickam A Munisankar S Bhootra Y Dolla C Thiruvengadam K Nutman TB. Metabolic Consequences of Concomitant Strongyloides Stercoralis Infection in Patients With Type 2 Diabetes Mellitus. Clin Infect Dis (2019) 69(4):697–704. 10.1093/cid/ciy935 30407548 PMC6669314

[51] Wolde M Berhe N Medhin G Chala F van Die I Tsegaye A. Inverse Associations of Schistosoma Mansoni Infection and Metabolic Syndromes in Humans: A Cross-Sectional Study in Northeast Ethiopia. Microbiol Insights (2019) 12:1178636119849934–1178636119849934. 10.1177/1178636119849934 31205419 PMC6537292

[52] Chen Y Lu J Huang Y Wang T Xu Y Xu M. Association of Previous Schistosome Infection With Diabetes and Metabolic Syndrome: A Cross-Sectional Study in Rural China. J Clin Endocrinol Metab (2013) 98(2):E283–7. 10.1210/jc.2012-2517 23275524

[53] Mohamed SMA Saber T Taha AA Roshdy HS Shahien NE. Relation Between Schistosome Past Infection and Metabolic Syndrome. J Egypt Soc Parasitol (2017) 47(1):137–43. 10.21608/jesp.2017.78014 30157342

[54] Htun NSN Odermatt P Müller I Yap P Steinmann C Schindler M. Association Between Gastrointestinal Tract Infections and Glycated Hemoglobin in School Children of Poor Neighborhoods in Port Elizabeth, South Africa. PLoS Negl Trop Dis (2018) 12(3):e0006332. 10.1371/journal.pntd.0006332 29543807 PMC5871004

[55] Htun NSN Odermatt P Paboriboune P Sayasone S Vongsakid M Phimolsarn-Nusith V. Association Between Helminth Infections and Diabetes Mellitus in Adults From the Lao People’s Democratic Republic: A Cross-Sectional Study. Infect Dis Poverty (2018) 7(1):105–5. 10.1186/s40249-018-0488-2 PMC6219195 30396368

[56] Sanya RE Webb EL Zziwa C Kizindo R Sewankambo M Tumusiime J. The Effect of Helminth Infections and Their Treatment on Metabolic Outcomes: Results of a Cluster-Randomised Trial. Clin Infect Dis (2019) 71:601–13. 10.1093/cid/ciz859 PMC7384320 31504336

[57] Cliffe LJ Humphreys NE Lane TE Potten CS Booth C Grencis RK. Accelerated Intestinal Epithelial Cell Turnover: A New Mechanism of Parasite Expulsion. Science (2005) 308(5727):1463–5. 10.1126/science.1108661 15933199

[58] Chen F Liu Z Wu W Rozo C Bowdridge S Millman A. An Essential Role for TH 2-Type Responses in Limiting Acute Tissue Damage During Experimental Helminth Infection. Nat Med (2012) 18(2):260–6. 10.1038/nm.2628 PMC3274634 22245779

[59] Johnston CJ McSorley HJ Anderton SM Wigmore SJ Maizels RM. Helminths and Immunological Tolerance. Transplantation (2014) 97(2):127. 10.1097/TP.0b013e3182a53f59 24025322 PMC3940291

[60] Godoy-Matos AF Júnior WSS Valerio CM. NAFLD as a Continuum: From Obesity to Metabolic Syndrome and Diabetes. Diabetol Metab Syndr (2020) 12(1):1–20. 10.1186/s13098-020-00570-y 32684985 PMC7359287

[61] Correale J Farez MF. The Impact of Parasite Infections on the Course of Multiple Sclerosis. J Neuroimmunol (2011) 233(1-2):6–11. 10.1016/j.jneuroim.2011.01.002 21277637

[62] Maizels RM McSorley HJ. Regulation of the Host Immune System by Helminth Parasites. J Allergy Clin Immunol (2016) 138(3):666–75. 10.1016/j.jaci.2016.07.007 PMC5010150 27476889

[63] Sartono E Kruize YC Kurniawan A van der Meide PH Partono F Maizels RM. Elevated Cellular Immune Responses and Interferon-γ Release After Long-Term Diethylcarbamazine Treatment of Patients With Human Lymphatic Filariasis. J Infect Dis (1995) 171(6):1683–7. 10.1093/infdis/171.6.1683 7769319

[64] Beknazarova M Whiley H Judd JA Shield J Page W Miller A. Argument for Inclusion of Strongyloidiasis in the Australian National Notifiable Disease List. Trop Med Infect Dis (2018) 3(2):61. 10.3390/tropicalmed3020061 PMC6073110 30274457

[65] Hyde Z Smith K Flicker L Atkinson D Almeida OP Lautenschlager NT. Mortality in a Cohort of Remote-Living Aboriginal Australians and Associated Factors. PLoS One (2018) 13(4):e0195030–e0195030. 10.1371/journal.pone.0195030 29621272 PMC5886486

[66] Anthony RM Rutitzky LI Urban JF Jr Stadecker RM Gause RM. Protective Immune Mechanisms in Helminth Infection. Nat Rev Immunol (2007) 7(12):975–87. 10.1038/nri2199 PMC2258092 18007680

[67] Gause WC Urban JF Jr. Stadecker MJ. The Immune Response to Parasitic Helminths: Insights From Murine Models. Trends Immunol (2003) 24(5):269–77. 10.1016/S1471-4906(03)00101-7 12738422

[68] Pearce E. Priming of the Immune Response by Schistosome Eggs. Parasite Immunol (2005) 27(7-8):265–70. 10.1111/j.1365-3024.2005.00765.x 16138847

[69] McSorley HJ Maizels RM. Helminth Infections and Host Immune Regulation. Clin Microbiol Rev (2012) 25(4):585–608. 10.1128/CMR.05040-11 23034321 PMC3485755

[70] Djuardi Y Wammes LJ Supali T Sartono E Yazdanbakhsh M. Immunological Footprint: The Development of a Child’s Immune System in Environments Rich in Microorganisms and Parasites. Parasitology (2011) 138(12):1508–18. 10.1017/S0031182011000588 21767432

[71] Mortimer K Brown A Feary J Jagger C Lewis S Antoniak M. Dose-Ranging Study for Trials of Therapeutic Infection With Necator Americanus in Humans. Am J Trop Med Hyg (2006) 75(5):914–20. 10.4269/ajtmh.2006.75.914 17123987

[72] de Ruiter K Tahapary DL Sartono E Soewondo P Supali T Smit JWA. Helminths, Hygiene Hypothesis and Type 2 Diabetes. Parasite Immunol (2017) 39(5). 10.1111/pim.12404 27925245

[73] Croese J Giacomin P Navarro S Clouston A McCann L Dougall A. Experimental Hookworm Infection and Gluten Microchallenge Promote Tolerance in Celiac Disease. J Allergy Clin Immunol (2015) 135(2):508–16.e5. 10.1016/j.jaci.2014.07.022 25248819

[74] Feary J Venn AJ Mortimer K Brown AP Hooi D Falcone FH. Experimental Hookworm Infection: A Randomized Placebo-Controlled Trial in Asthma. Clin Exp Allergy (2010) 40(2):299–306. 10.1111/j.1365-2222.2009.03433.x 20030661 PMC2814083

[75] McSorley HJ Gaze S Daveson J Jones D Anderson RP Clouston A. Suppression of Inflammatory Immune Responses in Celiac Disease by Experimental Hookworm Infection. PLoS One (2011) 6(9):e24092. 10.1371/journal.pone.0024092 21949691 PMC3174943

[76] Hotez PJ Brooker S Bethony JM Bottazzi ME Loukas A Xiao S. Hookworm Infection. N Engl J Med (2004) 351(8):799–807. 10.1056/NEJMra032492 15317893

[77] Olveda DU Li Y Olveda RM Lam AK Chau TN Harn DA. Bilharzia: Pathology, Diagnosis, Management and Control. Trop Med Surg (2013) 1(4):135–54. 10.4172/2329-9088.1000135 PMC4208666 25346933

[78] King CH Dickman K Tisch DJ. Reassessment of the Cost of Chronic Helmintic Infection: A Meta-Analysis of Disability-Related Outcomes in Endemic Schistosomiasis. Lancet (2005) 365(9470):1561–9. 10.1016/S0140-6736(05)66457-4 15866310

[79] Tang C-L Yu XH Li Y Zhang RH Xie J Liu ZM. Schistosoma Japonicum Soluble Egg Antigen Protects Against Type 2 Diabetes in Lepr (Db/Db) Mice by Enhancing Regulatory T Cells and Th2 Cytokines. Front Immunol (2019) 10:1471–1. 10.3389/fimmu.2019.01471 PMC6607994 31297120

[80] Zaccone P Burton O Miller N Jones FM Dunne DW Cooke A. Schistosoma Mansoni Egg Antigens Induce Treg That Participate in Diabetes Prevention in NOD Mice. Eur J Immunol (2009) 39(4):1098–107. 10.1002/eji.200838871 19291704

[81] Zaccone P Burton OT Gibbs S Miller N Jones FM Dunne DW. Immune Modulation by Schistosoma Mansoni Antigens in NOD Mice: Effects on Both Innate and Adaptive Immune Systems. J Biomed Biotechnol (2010) 2010:795210–0. 10.1155/2010/795210 PMC2830582 20204176

[82] Zaccone P Fehérvári Z Jones FM Sidobre S Kronenberg M Dunne DW. Schistosoma Mansoni Antigens Modulate the Activity of the Innate Immune Response and Prevent Onset of Type 1 Diabetes. Eur J Immunol (2003) 33(5):1439–49. 10.1002/eji.200323910 12731071

[83] Bhargava P Li C Stanya KJ Jacobi D Dai L Liu S. Immunomodulatory Glycan LNFPIII Alleviates Hepatosteatosis and Insulin Resistance Through Direct and Indirect Control of Metabolic Pathways. Nat Med (2012) 18(11):1665–72. 10.1038/nm.2962 PMC3493877 23104131

[84] Harnett MM Harnett W. Can Parasitic Worms Cure the Modern World’s Ills? Trends Parasitol (2017) 33(9):694–705. 10.1016/j.pt.2017.05.007 28606411

[85] Maizels RM Smits HH McSorley HJ. Modulation of Host Immunity by Helminths: The Expanding Repertoire of Parasite Effector Molecules. Immunity (2018) 49(5):801–18. 10.1016/j.immuni.2018.10.016 PMC6269126 30462997

