# Supplementary material for: The Impact of Helminth Infection on the Incidence of Metabolic Syndrome: A Systematic Review and Meta-Analysis
Source: Front Endocrinol (Lausanne). 2021 Aug 12;12:728396. doi: 10.3389/fendo.2021.728396 (PMC8397462; doi:10.3389/fendo.2021.728396)
Supplement: Supplementary file 1 [file Table_1.docx]

Supplementary Material

#### **Supplementary Table 1 |** Quality of studies determined using the JBI quality of assessment tool for analytical cross-sectional studies

| **Citation** | Q1. Were the criteria for inclusion in the sample clearly defined? | Q2. Were the study subjects and the setting described in detail? | Q3. Was the exposure measured in a valid and reliable way? | Q4. Were objective, standard criteria used for measurement of the condition? | Q5. Were confounding factors identified? | Q6. Were strategies to deal with confounding factors stated | Q7. Were the outcomes measured in a valid and reliable way? | Q8. Was appropriate statistical analysis used? |
| --- | --- | --- | --- | --- | --- | --- | --- | --- |
| Muthukumar R, *et al*. 2020. | Y | Y | Y | Y | Y | Y | Y | Y |
| Rajamanickam A, *et al.* 2019. | Y | Y | Y | Y | U | Y | Y | Y |
| Wiria AE, *et al*. 2015. | U | Y | Y | Y | Y | Y | Y | Y |
| Wolde M, *et al.* 2019. | Y | Y | Y | Y | Y | Y | Y | Y |
| Sanya RE*, et al*. 2019. | Y | Y | Y | Y | Y | Y | Y | Y |
| Hays R, *et al*. 2015. | Y | Y | Y | Y | Y | Y | Y | Y |
| Htun NSN, *et al.* 2018. | Y | Y | Y | Y | Y | Y | Y | Y |
| Htun NSN, *et al.* 2018. | Y | Y | Y | U | Y | Y | Y | Y |
| Mohamed SMA, *et al.*2017. | Y | Y | Y | Y | Y | Y | Y | Y |
| Chen Y, *et al.* 2013. | Y | Y | Y | Y | Y | Y | Y | Y |
| % | 90.0 | 100.0 | 100.0 | 90.0 | 90.0 | 100.0 | 100.0 | 100.0 |

#### **Supplementary Table 2 |** Quality of studies determined using the JBI quality of assessment tool for case control studies

| **Citation** | Q1. Were the groups comparable other than the presence of disease in cases or the absence of disease in controls? | Q2. Were cases and controls matched appropriately? | Q3. Were the same criteria used for identification of cases and controls? | Q4. Was exposure measured in a standard, valid and reliable way? | Q5. Was exposure measured in the same way for cases and controls? | Q6. Were confounding factors identified? | Q7. Were strategies to deal with confounding factors stated? | Q8. Were outcomes assessed in a standard, valid and reliable way for cases and controls? | Q9. Was the exposure period of interest long enough to be meaningful? | Q10. Was appropriate statistical analysis used? |
| --- | --- | --- | --- | --- | --- | --- | --- | --- | --- | --- |
| Duan Q, *et al.* 2018. | Y | Y | Y | U | U | N | N/A | U | Y | Y |
| McGuire E, *et al.* 2019. | Y | Y | Y | Y | Y | Y | Y | Y | Y | Y |
| % | 100.0 | 100.0 | 100.0 | 50.0 | 50.0 | 50.0 | 50.0 | 50.0 | 100.0 | 100.0 |

#### **Supplementary Table 3 |** Quality of studies determined using the JBI quality of assessment tool for cohort studies

| **Citation** | Q1. Were the two groups similar and recruited from the same population? | Q2. Were the exposures measured similarly to assign people to both exposed and unexposed groups? | Q3. Was the exposure measured in a valid and reliable way? | Q4. Were confounding factors identified? | Q5. Were strategies to deal with confounding factors stated? | Q6. Were the groups/participants free of the outcome at the start of the study (or at the moment of exposure)? | Q7. Were the outcomes measured in a valid and reliable way? | Q8. Was the follow up time reported and sufficient to be long enough for outcomes to occur? | Q9. Was follow up complete, and if not, were the reasons to loss to follow up described and explored? | Q10. Were strategies to address incomplete follow up utilized? | Q11. Was appropriate statistical analysis used? |
| --- | --- | --- | --- | --- | --- | --- | --- | --- | --- | --- | --- |
| Shen S-W, *et al.* 2015. | Y | Y | Y | Y | Y | N/A | Y | Y | N/A | N/A | Y |
| Hays R, *et al* 2015. | Y | Y | Y | Y | Y | N/A | Y | N/A | N/A | N/A | Y |
| % | 100.0 | 100.0 | 100.0 | 100.0 | 100.0 | 0.0 | 100.0 | 50.0 | 0.0 | 0.0 | 100.0 |

| **Authors**  **Supplementary Table 4 \|** Study characteristics and primary results | **Year** | **Study type** | **Population details** | **Method of diagnosis** | **Helminth** | | **Measure of glucose homeastasis** | | | | **Quility score** |
| --- | --- | --- | --- | --- | --- | --- | --- | --- | --- | --- | --- |
|  |  |  |  |  |  |  | **FBG (mmol/L)** | **HbA1c ≥ 6.5 (%)** | **Fasting serum insulin (pmol/L)** | **HOMA-IR** |  |
| Chen *et al.*  China | 2013 | Cross-sectional | Sample size - 9939, age ≥ 40 years | Cross valiation of self reported PSI with registry data generated by the local government during the screening conducted in 1989 | Schistosoma spp. | INF | 5.5±1.43 | 5.8 | - | 1.2 | 16 |
|  |  |  |  |  |  | Ctrl | 5.7±1.5 | 5.92 | - | 1.73 |  |
| Duan *et al.*  China | 2018 | Case control | Sample size - 3981, average age - 50.63 | Unreported | *S. japonicum* | INF | 5.33 | - | - | - | 13 |
|  |  |  |  |  |  | Ctrl | 5.56 | - | - | - |  |
| Hays *et al.* (1) Australia | 2015 | Cross-sectional | Sample size - 259, average age 43.4 years | Serology | *S. stercoralis* | INF | - | - | - | - | 16 |
|  |  |  |  |  |  | Ctrl | - | - | - | - |  |

| Hays *et al.* (2)  Australia | 2015 | Cohort | Sample size - 259, average age 43.4 years | Serology | *S. stercoralis* | | INF | 8.2±2.8 | - | - | - | 18 |
| --- | --- | --- | --- | --- | --- | --- | --- | --- | --- | --- | --- | --- |
|  |  |  |  |  |  |  | Ctrl | 5.9±0.4 | - | - | - |  |
| Htun *et al*. (1)  South Africa | 2018 | Cross-sectional | Sample size 842, age 9-14 years | Stool sample assessed via microscopy | Multiple | *A. lumbricoides* | INF | - | 5.7 | - | - | 16 |
|  |  |  |  |  |  |  | Ctrl | - | 5.8 | - | - |  |
|  |  |  |  |  |  | *T. trichura* | INF | - | 5.7 | - | - |  |
|  |  |  |  |  |  |  | Ctrl | - | 5.8 | - | - |  |
|  |  |  |  |  |  | *E. vermicularis* | INF | - | 5.7 | - | - |  |
|  |  |  |  |  |  |  | Ctrl | - | 5.8 | - | - |  |
|  |  |  |  |  |  | *S. mansoni* | INF | - | 5.9 | - | - |  |
|  |  |  |  |  |  |  | Ctrl | - | 5.8 | - | - |  |
|  |  |  |  |  |  | *S. haematobium* | INF | - | 5.8 | - | - |  |
|  |  |  |  |  |  |  | Ctrl | - | 5.8 | - | - |  |
| Htun *et al*. (2)  Lao People's Democratic Republic | 2018 | Cross-sectional | Sample size - 1600, age ≥ 35 years | Stool sample assessed via microscopy | Multiple | | INF | - | - | - | - | 16 |
|  |  |  |  |  |  |  | Ctrl | - | - | - | - |  |

| McGuire *et a*l. England | 2019 | Case control | Sample size - 532, average age 57 years | Serology | *S. stercoralis* | INF | - | - | - | - | 20 |
| --- | --- | --- | --- | --- | --- | --- | --- | --- | --- | --- | --- |
|  |  |  |  |  |  | Ctrl | - | - | - | - |  |
| Mohamed *et al.* Egypt | 2017 | Cross-sectional | Sample size - 574, age 41-70 years | Stool sample assessed via microscopy | Schistosoma spp. | INF | 5.9±1.3 | - | - | - | 16 |
|  |  |  |  |  |  | Ctrl | 6±1.31.5 | - | - | - |  |
| Muthukumar *et al.* Thailand | 2020 | Cross-sectional | Sample size - 400, age ≥ 30 years | Stool sample assessed via microscopy | *O. viverrini* | INF | - | 7.0 | - | - | 16 |
|  |  |  |  |  |  | Ctrl | - | 13.5 | - | - |  |
| Rajamanickam *et al.* India | 2018 | Cross-sectional | Sample size - 158, ages 18-75 | Stool microscopy and serology | *S. stercoralis* | INF | - | 8.75±1.8 | - | - | 15 |
|  |  |  |  |  |  | Ctrl | - | 8.9±1.5 | - | - |  |
| Shen *et al.* China | 2015 | Cohort study | Sample size - 1597, age ≥ 45 years | Stool sample assessed via microscopy | *S. japonicum* | INF | 6.21±1.6 | - | - | - | 19 |
|  |  |  |  |  |  | Ctrl | 6.38±1.5 | - | - | - |  |

| Sanya *et al.*  Uganda | 2019 | (Randomised controlled trial) Cross-sectional | Sample size - 1898, age ≥ 10 years | Stool samples assessed via microscopy and/or PCR | Multiple | *S. mansoni* | INF | 4.74 | 5.0 | - | 1.69 | 16 |
| --- | --- | --- | --- | --- | --- | --- | --- | --- | --- | --- | --- | --- |
|  |  |  |  |  |  |  | Ctrl | 4.88 | 5.0 | - | 1.9 |  |
|  |  |  |  |  |  | Hookworm | INF | 4.67 | 5.1 | - | 1.76 |  |
|  |  |  |  |  |  |  | Ctrl | 4.82 | 5.0 | - | 1.83 |  |
|  |  |  |  |  |  | *S. stercoralis* | INF | 4.84 | 5.1 | - | 1.25 |  |
|  |  |  |  |  |  |  | Ctrl | 4.81 | 5.0 | - | 1.89 |  |
|  |  |  |  |  |  | Any | INF | 4.82 | 5.0 | - | 1.9 |  |
|  |  |  |  |  |  |  | Ctrl | 4.92 | 5.0 | - | 2.12 |  |
| Wiria *et al.* Indonesia | 2015 | Cross-sectional | Sample size - 464, age ≥ 18 years | Stool samples assessed via microscopy for *T. trichuris,* PCR for the others | Multiple | | INF | 5.88±1.5 | - | 45 | 0.81±0.86 | 15 |
|  |  |  |  |  |  |  | Ctrl | 5.92±1.6 | - | 49.5 | 0.97±0.84 |  |
| Wolde *et al.* Ethiopia | 2019 | Cross-sectional | Sample size 181, age ≥ 18 years | Stool sample assessed via microscopy | *S. mansoni* | | INF | 5.22 | - | 15.2 (umol/ml) | - | 16 |
|  |  |  |  |  |  |  | Ctrl | 6.34 | - | 12 (umol/ml) | - |  |

**
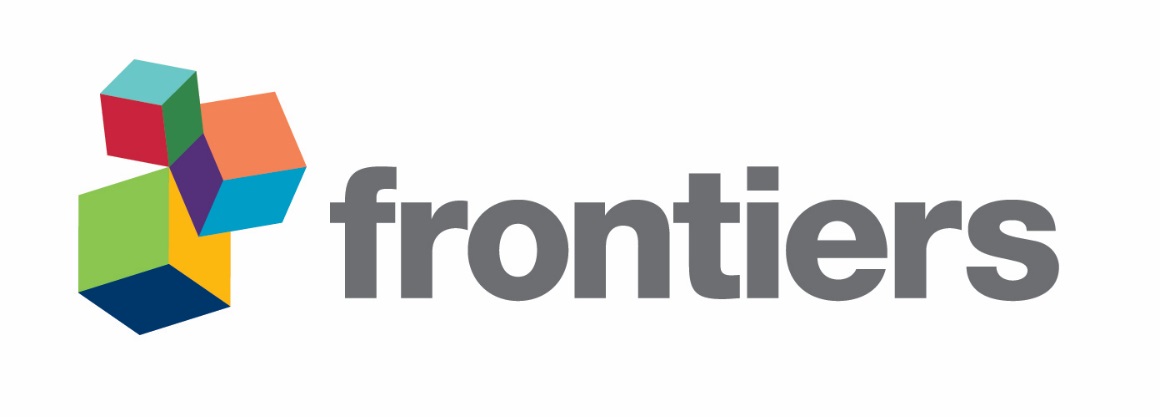
**
